# Developmental Fluoride Neurotoxicity: Clinical Importance versus Statistical Significance

**DOI:** 10.1289/ehp.1206192

**Published:** 2013-03-01

**Authors:** Siamak Sabour, Zahra Ghorbani

**Affiliations:** School of Dentistry, Shahid Beheshti University of Medical Sciences, Tehran, Iran, E-mail: s.sabour@sbmu.ac.ir

We were interested to read the article by Choi et al. (2012), who investigated the effects of increased fluoride exposure and delayed neurobehavioral development by reviewing published studies and performing a meta-analysis. Of the 39 studies identified, the authors considered 27 to be eligible. Choi et al. reported a mean difference in IQ (intelligence quotient) score between exposed and reference populations of –0.4 (95% confidence interval: –0.5, –0.3) using a random-effects model. Thus, children in high-fluoride areas had significantly lower IQ scores than those who lived in low-fluoride areas.

Even if we ignore the weaknesses of the study (Choi et al. 2012), including a lack of individual-level information and the high probability of confounding because the authors did not adjust for covariates, a difference of 0.4 in mean IQ is clinically negligible (Jeckel et al. 2007; Rothman et al. 2008; Szklo and Nieto 2007) even though it was statistically significant. In general, clinical importance takes priority over statistical significance. The *p*-value can easily change from significant to nonsignificant because of sample size or the mean difference and standard deviation of the variable in the study population (Jeckel et al. 2007; Rothman et al. 2008; Szklo and Nieto 2007). As Choi et al. (2012) pointed out in their conclusion, there is a “possibility of an adverse effect of high fluoride exposure on children’s neurodevelopment.” Such a conclusion can be considered an ecological fallacy, which can easily lead to misinterpretation of the results. It is important to know that statistics cannot provide a simple substitute for clinical judgment (Jeckel et al. 2007; Rothman et al. 2008; Szklo and Nieto 2007).
